# Structural Health Monitoring with Sensor Data and Cosine Similarity for Multi-Damages

**DOI:** 10.3390/s19143047

**Published:** 2019-07-10

**Authors:** Byungmo Kim, Cheonhong Min, Hyungwoo Kim, Sugil Cho, Jaewon Oh, Seung-Hyun Ha, Jin-hak Yi

**Affiliations:** 1 Department of Convergence Study on the Ocean Science and Technology, Ocean Science and Technology School of Korea Maritime and Ocean University, Busan 49112, Korea; 2 Offshore Industries R&BD Center, Korea Research Institute of Ships & Ocean Engineering (KRISO), Geoje 53201, Korea; 3 Department of Ocean Engineering, Korea Maritime and Ocean University, Busan 49112, Korea; 4 Coastal Development and Ocean Energy Research Center, Korea Institute of Ocean Science & Technology, Busan 49111, Korea

**Keywords:** damage detection, cosine similarity, structural health monitoring, system identification, structural integrity assessment

## Abstract

There is a large risk of damage, triggered by harsh ocean environments, associated with offshore structures, so structural health monitoring plays an important role in preventing the occurrence of critical and global structural failure from such damage. However, obstacles, such as applicability in the field and increasing calculation costs with increasing structural complexity, remain for real-time structure monitoring offshore. Therefore, this study proposes the comparison of cosine similarity with sensor data to overcome such challenges. As the comparison target, this method uses the rate of changes of natural frequencies before and after the occurrence of various damage scenarios, including not only single but multiple damages, which are organized by the experiment technique design. The comparison method alerts to the occurrence of damage using a normalized warning index, which enables workers to manage the risk of damage. By comparison, moreover, the case most similar with the current status is directly figured out without any additional analysis between monitoring and damage identification, which renders the damage identification process simpler. Plus, the averaged rate of errors in detection is suggested to evaluate the damage level more precisely, if needed. Therefore, this method contributes to the application of real-time structural health monitoring for offshore structures by providing an approach to improve the usability of the proposed technique.

## 1. Introduction

**Figure 1:**
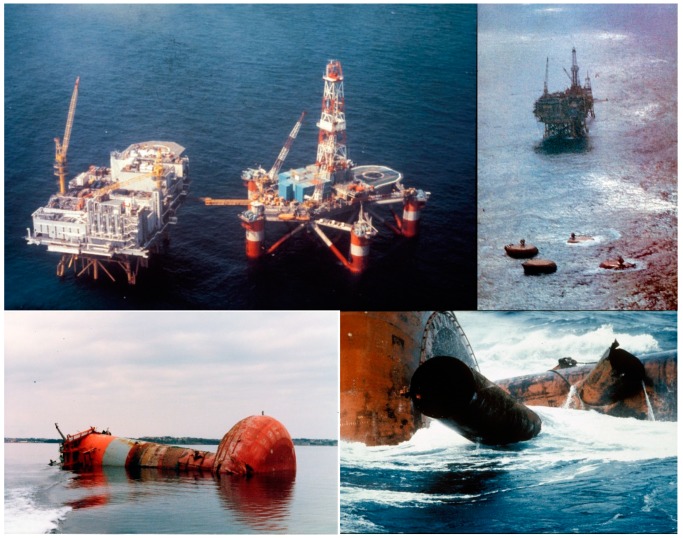
Alexander L. Kielland and capsize of it [2].

In the history of offshore operations, various hazards have led to accidents with severe consequences, ranging from oil spills to the death of people. Among them, structural problems, such as damage and failure triggered by fatigue, corrosion, wear, etc., are considered as one of the main causes. For example, it is officially known that the root cause of the capsizing of the Alexander L. Kielland platform was a crack on a brace induced by fatigue [1], as shown in Figure 1. On this point, structural health monitoring (SHM) and damage detection can be an effective solution to prevent such hazardous events.

Moreover, an innovative trial for an unmanned and automated system in current offshore development, for instance, the Valemon and K-15 platform [3], would obviously increase the necessity of an automatic SHM based on real-time field monitoring during operation. In fact, accurate and time-continuous structural integrity monitoring technology will be needed to support immediate and correct decision-making of the new system since the unmanned condition would make action delayed in any unexpected situation, compared to the conventional management by workers residing on the platform.

On the other hand, SHM technology has not yet reached an adequate stage for complete real-time integrity diagnosis of an offshore structure [4], although this research field is growing. In fact, SHM has developed a wide range of applications in industrial fields, e.g., buildings, bridges, wind turbines, aircrafts, and so on. For instance, a new approach to detect the tendon damage of pre-stressed concrete (PSC) bridges based on a convolutional auto-encoder has recently been proposed, and it is applicable for multiple vehicles under severe damage conditions [5]. One very popular approach to traditional SHM is vibration-based damage detection [6] using modal parameters, such as natural frequencies and mode shapes. In recent years, the classical technique has evolved into statistical time series SHM methods [7], and is further faced with the beginning of the big data, which is still quite challenging [8], but may be more applicable to real-time SHM during operation.

For big data relevant SHM, several studies have been performed lately. Above all, the concept and procedure of SHM were comparatively analyzed in aspects of the big data, and the process of the big data relevant SHM was summarized, including data cleansing, dimensionality reduction, data fusion and feature extraction, feature classification, outlier detections, and prediction [9]. Cremona and Santos categorized SHM into two approaches, inverse and forward strategies [10]. While the former was introduced as the most popular method, today, based on model updating or system identification (SI), the latter was mentioned as the opposite of the previous approach because it only requires measured data but not any numerical or analytical model. Therefore, it was considered that the data-driven method is very prospective since it would be more suitable for the real-time analysis of large-scale structures for SHM as well as being more involved with the philosophy of big data [10]. As an example of relevant research, a fully data-driven SHM method for bridges was recently presented, which applied tensor analysis techniques to acquire multi-dimensional sensor data for data fusion and feature extraction [11].

From a technical point of view based on data science, meanwhile, Matarazzo, et al. showed that the calculation accuracy of modal parameters and damage diagnosis was not improved proportionally with the number of sensors [12]. As an example, it was shown that an 8 and 10 sensor scheme with proper SHM techniques is enough to identify a simple beam’s modal properties and structural damages, respectively [12]. Conversely speaking, however, it could be unfavorable for complicated structures. In detail, the sizes of big data for SHM can be exorbitantly large depending on the number of members of the structure because output-only SI, i.e., operational modal analysis (OMA), and algorithms generally require computational costs that increase cubically and linearly with the number of sensor channels and samples, respectively. Moreover, most offshore structures consist of lots of members, which means that the number of required sensors would be a lot as well. Consequently, it causes huge computing costs to increase proportionally with the structural complexity.

To overcome this challenge, two different approaches are possible. One of them is to improve the efficiency of data driven SHM, for example, to make data analytic techniques consume less time. Another way is a hybrid method of the conventional inverse SHM and data processing methods to make the procedure of damage detection more suited for real-time SHM. As for the first, there are some relevant research studies were performed. Cai and Mahadevan proposed the MapReduce technique for image data-based online SHM [13]. Moreover, Salehi, et al. showed that it is possible to detect damage from discretized, noisy, and delayed signals from wireless sensor networks (WSNs) by using artificial intelligence, particularly pattern recognition and machine learning, with a probabilistic approach [14]. It is obvious that this new data driven SHM would broaden the applicability of real-time SHM to real fields, but it thus far, it has not been demonstrated that the technique works well for multiple damages. Further, Sen, et al. proposed an effective data driven SHM method to detect small local damage, like cracks and corrosions, on a pipe using semi-supervised and supervised machine learning [15]. This is helpful in on-the-spot inspection over several candidates of places where damages are likely to occur but would not be suitable for global SHM for offshore structures. Regarding the hybrid approach, on the other hand, new trials in recent days are briefly summarized. Above all, the fourth-order voltage statistical moments (FVSMs) method based on piezoelectric measurement with a spectral element model (SEM) was proposed [16]. The method successfully identified additional mass as well as crack damages from voltage output in the time domain. However, a total computation cost of almost 5000 s, including a differential evolution algorithm (DEA) to find the optimal solution for damages, is likely to be relatively huge for real-time SHM. In addition, a damage identification method using only the first vibration mode data was proposed by Jahangiri et al., and the core idea of it is that it harnesses the relative discrepancy function (RDF) comprised of relative differences of the natural frequency and mode shape vector as the object function to identify optimal damage scenarios [17]. It was numerically validated that the method could detect single and multiple damages on several truss and frame structures as examples. However, experiments are essentially needed to assure whether the algorithm is applicable to real structures. Lastly, libraries of normal and degradation patterns from a large available historical database were used to evaluate similarities for fault detection [18]. To be more specific, this method basically came from the non-parametric approach for pattern analysis and prediction. The merit of this is, hence, that it is less cost-consuming in damage evaluation than the output-only SI if there are sufficient data accumulated from lots of identical objects during past operation. This manner can be categorized as a kind of similarity-based method, which is directly involved with the main topic of this paper. In the case of the offshore industry, however, almost every offshore structure is customized for their mission and environmental condition in the design and manufacturing process, which in turn makes the structural characteristics of individuals very distinctive. This makes accumulated data from other past cases less applicable. Plus, most offshore fields are far from onshore networks. Therefore, an advantage of an online network system would not be applied to manage massive amounts of data.

In order to solve these obstacles, Min et al. proposed a cosine similarity-based damage detection method for a single damage [19]. The method estimates cosine similarity compared to a database consisting of sensitivities of rates of change of natural frequencies corresponding to decreases of the elastic modulus of each element. However, the ability to figure out multi-damages is required for damage detection, with a view to application for real structures. Therefore, this paper proposes an improved method capable of monitoring multiple damages simultaneously. In detail, rates of change of natural frequencies themselves under multiple damage conditions are comprised of the database called a damage estimation matrix for similarity comparison, instead of their sensitivities for an individual element, and the rate of errors, on average, is suggested for more accurate identification of damage severities. The database is built from a well-designed numerical model with appropriate design of experiment (DOE) methods but not from historical databases. Ultimately, this method pursues a more convenient estimation for real-time damage evaluation in offshore fields by utilizing cosine similarity with the operational modal analysis technique rather than data-driven SHM. Consequently, the manner proposed in this paper provides candidates of possible damage scenarios inclusive of severities and locations in the order of similarity from simple and fast calculation. Thus, this enables anyone in the field to take responsibility to recognize and treat potential risks for damage. Therefore, this approach also contributes to grafting real-time SHM onto remote offshore operations, where it is difficult to monitor structural integrity in traditional ways due to the poor accessibility of SHM experts, as well as the capability of multiple damage detection.

## 2. Methodology

In general, SHM starts from measurement and monitoring so relevant topics have been actively studied since not only are there many important issues in the step of signal processing, such as noises, delay of signals, and missed signals, but also the types and characteristics of the measurement system affect subsequent steps of SHM. For example, the type of sensors for SHM used in aircraft, ranging from fiber optic sensor, piezoelectric, electro-magnetic, and laser sensors to nanomaterials, and their monitoring principles were comprehensively enumerated and, in particular, applications of piezoelectric transducer-based SHM for aircrafts were overviewed [20]. The applicability of piezoelectric wafer active sensors (PWASs) under severe temperature and radiation conditions for SHM was thoroughly proved [21]. The sensing range of a piezoelectric interface-based impedance monitoring system for SHM was numerically and experimentally figured out on plate-type structures [22]. In addition, Radzieński et al. utilized a laser vibrometer to detect hidden damage in composite plates and it was verified that the material independency of the non-destructive method is an advantage of the technique [23]. Regarding a pipe as an example of the structural types widely used for offshore and aerospace structures, additionally, Wang et al. briefly summed up recent research trends and issues for sensing and data processing, for instance, saturation and electromagnetic interference of piezoelectric transducers as well as wiring [24]. Fiber Bragg grating (FBG) sensors were considered as the alternative way instead of electronic sensors.

**Figure 2:**
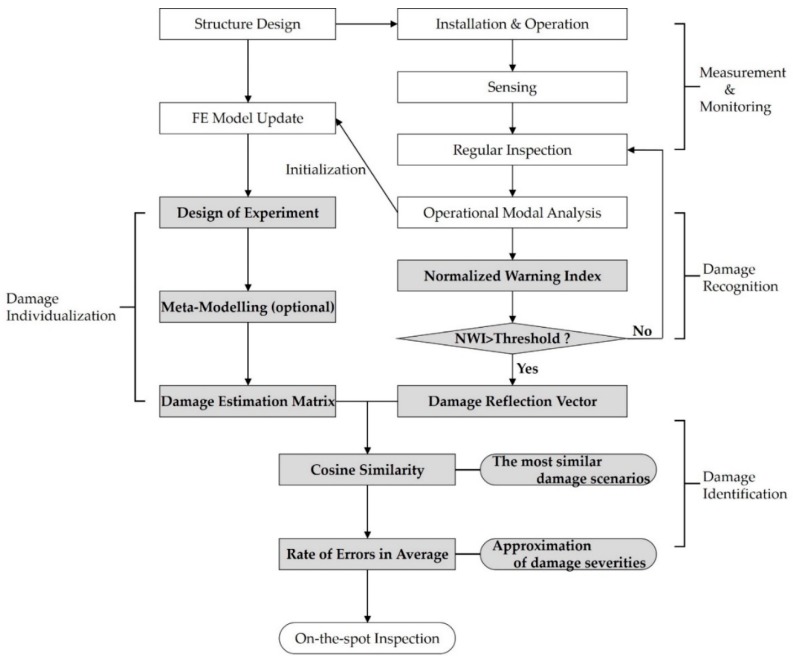
Entire flowchart of cosine similarity-based SHM.

On the other hand, this study focuses on subsequent steps of the measurement and monitoring to make the whole monitoring system more suitable for inspectors and operators offshore in recognizing and identifying the occurrence of multiple damages regardless of the type of sensors. The entire procedure can be summarized as shown in Figure 2. The initialization of the FE model update is literally performed using the result of OMA at the first inspection. In particular, the shaded parts are the main scope in this study.

### 2.1. Damage Individualization: Damage Estimation Vector and Matrix

There are various types of damages, ranging from corrosion and degradation to cracking, and all types of damages cause a loss of mass, change of material properties, and/or some kind of irreversible deformation. The most popular way to describe them, in general, is to regard them as a decrease of stiffness, in particular the elastic modulus. Thus, it is noted that this paper investigates it as well.

With a view to damage detection using similarity, comparison targets are needed, which are correctly matched to how much a structure is damaged, and various types of responses and structural characteristics may be theoretically available. Regardless of what is utilized for the comparison, however, distinct features of an offshore structure rising from customization in design, and large and small causes in manufacturing and installation may give rise to an uncertainty of the relation between the targets and damage. Thus, the FE model must be necessarily updated to be fitted to an initial state and subsequently, a database on the relation of the targets customized for the structure under discretized damage conditions should be built. In this paper, this process is deemed as damage individualization.

Basically, structural dynamic properties, like natural frequencies or mode shapes, reflect the effect of structural damage. This is fundamentally because damage leads to a change of the structure, and subsequently to structural properties, such as stiffness and/or mass, and in turn, the dynamic properties. In this paper, therefore, natural frequencies, one of the representative dynamic characteristics, are used to evaluate damage. Particularly, the rate of change in a natural frequency before and after damage of a pre-updated FE model is considered as Equation (1), and a vector comprised of them from the *1*st to *n*th mode, which is named the damage estimation vector (DEV) in this study, is utilized as a similarity comparison target, as shown in Equation (2). For accurate damage identification, it is required the DEVs are prepared under a variety of damage conditions as the comparison targets. Above all, lots of damage cases are designed depending on damage locations and severities based on DOE. Then, modal analysis is carried out in accordance with DOE, and natural frequencies under the damage scenarios are acquired. Afterwards, the DEVs are calculated from the natural frequencies and constructed as a matrix named the damage estimation matrix (DEM), as shown in Equation (3). In other words, the DEM is a matrix made up of DEVs and plays a role in a sort of damage indices:

(1) $$z_{i,j}=\frac{f_{i,j}^{*}-f_{j}}{f_{j}},$$

(2) $$s_{i}={\left|\begin{array}{cc} \begin{array}{ccc} z_{i,1} & z_{i,2} & \cdots \end{array} & \begin{array}{ccc} z_{i,j} & \cdots & z_{i,n} \end{array} \end{array}\right|}^{T},$$

(3) $$S={\left|\begin{array}{cc} \begin{array}{cc} s_{1} & s_{2} \end{array} & \begin{array}{cc} \cdots & s_{m} \end{array} \end{array}\right|}^{T}=\left[\begin{array}{cc} \begin{array}{cc} z_{1,1} & z_{1,2}\\ z_{2,1} & z_{2,2} \end{array} & \begin{array}{cc} \cdots & z_{1,n}\\ \cdots & z_{2,n} \end{array}\\ \begin{array}{cc} \vdots & \vdots \\ z_{m,1} & z_{m,2} \end{array} & \begin{array}{cc} z_{i,j} & \vdots \\ \cdots & z_{m,n} \end{array} \end{array}\right],$$

where *i* and *m* is an arbitrary index number and the total number of the designed damage conditions respectively; *j* and *n* is an arbitrary and maximum available mode number, respectively; *f_j_* is the *j*th natural frequency of the undamaged FE model; $f_{i,j}^{*}$ is the *j*th natural frequency of the FE model in the *i*th damage case; *z_i,j_* is the rate of change of the *j*th natural frequency under the *i*th damage condition; ***s_i_*** is the DEV for the *i*th damage case; and ***S*** is the DEM for all the designed damage conditions.

### 2.2. Damage Recognition: Normalized Warning Index and Damage Reflection Vector

During operation, a maintenance system of an offshore structure monitors any kind of structural response, such as strain or acceleration. Regardless of whether it is monitored steadily or not and regardless of the physical response measured, the collected data is inspected by the OMA techniques, generally at regular time intervals, and natural frequencies are also cumulatively acquired, as shown in Equation (4). Then, the rate of change in natural frequencies between two sequential inspection cycles can be calculated by Equation (5), which is used as a warning index (WI) in this study.

The measured data always includes uncertainties and variations due to a lot of causes, e.g., noise or change of external factors, so the data and the WI can be thought of as a statistical random variable. As mentioned before on the relation with the damage and structural dynamic properties, it is evident that the damage results in unusual natural frequencies, which means that the occurrence of damage makes the WI distinguishable compared to other previous normal WI values. In other words, from a statistical point of view, the WI under a damaged condition is much more likely to be in the tail of the probability distribution of the WIs as the random variable. Based on such an approach, the mean value and standard deviation of the accumulated WIs until the *p*th inspection are calculated, and then a normalized warning index (NWI) is computed by subtracting the mean to the *p*th WI then dividing it by the standard deviation, as shown in Equation (6). Finally, the occurrence of damage is recognized by comparing the NWI with a damage threshold, which is an appropriate value of the random variable corresponding to the NWI. As an example, assuming that the random variable follows the standard normal distribution and the damage threshold is 3, 2, and 1, the possibilities of a damage corresponding to the threshold values is 99.87%, 97.7%, and 84%, respectively, referring to the standard normal distribution table. Therefore, damage would be perceived if the NWI is larger than the threshold value at the *d*th inspection, and in turn, the rates of changes in natural frequencies between the *d*−1th and *d*th inspections are vectorized, named the damage reflection vector (DRV) in this study, as shown in Equation (7):

(4) $$D={\left|\begin{array}{cc} \begin{array}{ccc} d_{1} & d_{2} & \cdots \end{array} & \begin{array}{ccc} d_{k} & \cdots & d_{p} \end{array} \end{array}\right|}^{T}=\left[\begin{array}{cc} \begin{array}{cc} f_{1}^{1} & f_{2}^{1}\\ f_{1}^{2} & f_{2}^{2} \end{array} & \begin{array}{cc} \cdots & f_{n}^{1}\\ \cdots & f_{n}^{2} \end{array}\\ \begin{array}{cc} \vdots & \vdots \\ f_{1}^{p} & f_{2}^{p} \end{array} & \begin{array}{cc} f_{j}^{k} & \vdots \\ \cdots & f_{n}^{p} \end{array} \end{array}\right],$$

(5) $$WI_{j}^{p}=\frac{f_{j}^{p}-f_{j}^{p-1}}{f_{j}^{p-1}},$$

(6) $$NWI_{j}^{p}=\frac{WI_{j}^{p}-\mu_{j}^{p-1}}{\sigma_{j}^{p-1}},$$

(7) $$h={\left|\begin{array}{cc} \begin{array}{cc} WI_{1}^{d} & WI_{2}^{d} \end{array} & \begin{array}{cc} \cdots & WI_{n}^{d} \end{array} \end{array}\right|}^{T}={\left|\begin{array}{cc} \begin{array}{cc} \frac{f_{1}^{d}-f_{1}^{d-1}}{f_{1}^{d-1}} & \frac{f_{2}^{d}-f_{2}^{d-1}}{f_{2}^{d-1}} \end{array} & \begin{array}{cc} \cdots & \frac{f_{n}^{d}-f_{n}^{d-1}}{f_{n}^{d-1}} \end{array} \end{array}\right|}^{T},$$

where *k* and *p* is an arbitrary cycle number and the most recent cycle number of the inspections, respectively; *f_j_^k^* is the *j*th natural frequency at the *k*th inspection cycle; ***d_k_*** is the vector of the natural frequencies at the *k*th inspection cycle; ***D*** is a matrix consisting of all the natural frequencies by the most recent inspection; *WI_j_^p^* is the warning index of the *j*th natural frequency at the *p*th inspection cycle; *d* is the inspection cycle number when a damage is recognized; and ***h*** is the DRV at the time.

There are two benefits in this way. The first is that damage recognition can be done only based on the tendency of the measured data. That is, a baseline FE model is not essential, at least to be aware of the occurrence of damage. The second merit is, therefore, that it allows this method to be more broadly applied to real structures. This is because damage can be identified even in the case that there is no accurate baseline FE model due to an unclear structural state. The fact of the matter is, moreover, a lot of structures are old and have not been thoroughly monitored for maintenance. Therefore, this method could be extensively utilized in such cases, although it would be difficult to identify exact places and levels of damages.

### 2.3. Damage Identification: Cosine Similarity and Rate of Errors in Detection

Once occurrence of damage is recognized, its locations and severities are identified as soon and as accurately as possible. Basically, if the damage is exactly the same as one of the damage scenarios, the DEV and DRV will be identical as well. That is, the similarity between the predefined DEM and DRV can be used as a key parameter for damage identification. There are various similarity measures, and Cha comprehensively classified them, ranging from an intersection family and an inner product family to a fidelity family for comparison of probability density functions. In particular, the inner product family embraces the inner product, harmonic mean, cosine, Humar–Hassebrook, and Jaccard, and Dice coefficient [25]. These techniques have also been extensively applied to data mining in recent years. Especially, the cosine similarity is one of the most popular methods to analyze the features and tendency of vectorized data based on directional similarity [26]. Mathematically, it is calculated by normalizing the dot product of two vectors, so the calculation is simple and intuitive. Plus, it also provides a result listed in the order of similarity, i.e., ranking [27]. Hence, it was employed in this study for similarity assessment of the DEM and DRV as shown in Equation (8). For instance, if fortunately, the cosine similarity value is exactly 1, the angle between two vectors is 0, meaning that they have the same direction. Therefore, accurate damage locations and severities might be directly identified. Even if it does not, the method also helps an inspector to know which damage conditions among a variety of cases in the DEM are more similar with the current structural status than the others. Thus, the most possible damage scenarios would be found. Thus, this approach maximizes the usability of damage detection in operation and consequently improves the convenience of workers and inspectors in the field, as well:

(8) $$CS_{i}=\frac{s_{i}\cdot h}{\left\|s_{i}\|\|h\right\|}=\frac{\sum_{j=1}^{n}z_{i,j}WI_{j}^{d}}{\sqrt{\sum_{j=1}^{n}{\left(z_{i,j}\right)}^{2}}\sqrt{\sum_{j=1}^{n}{\left(WI_{j}^{d}\right)}^{2}}},$$

where *CS_i_* is the cosine similarity value of the *i*th DEV for the damage.

**Figure 3:**
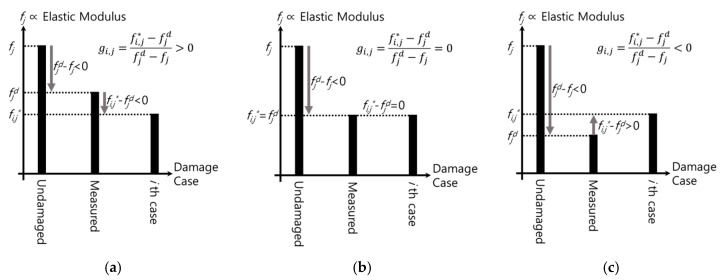
The concept of the rate of errors in detection (RED): (**a**) *g_i,j_* > 0; (**b**) *g_i,j_* = 0; (**c**) *g_i,j_* < 0.

On the other hand, the cosine value being exactly 1 is not a frequent occurrence because the DEM includes not continuous but discretized, i.e., finite, damage cases even though the matrix is well organized by the DOE technique. As circumstances require, hence, it would be additionally needed to more precisely assess how much the elastic modulus decreases. For such needs, the real value of the elastic modulus under the damaged condition is approximately estimated based on previously defined parameters. To explain, the error rate of the DEV for the DRV, termed as the rate of errors in detection (RED) in this paper, is proposed as Equation (9). The element of it for an arbitrary *j*th mode is calculated under the assumption that the natural frequencies inspected before the damage are the same as those of the undamaged structure, i.e., *f_j_^d−^*^1^ = *f_j_*, as shown in Equation (10). In detail, the error rate, *g_i,j_*, for the *j*th mode indicates, as shown in Figure 3, the relation between the unknown elastic modulus under damage and the elastic modulus of the comparison target case. This relationship also exists in all available modes, so, as shown in Equation (11), the average of the elements of the vector, ***g_i_***, as a representative parameter named the rate of errors on average (REA) in this paper, is used to estimate the reduced value of the Young’s modulus. More specifically, the top-ranked damage scenario in the similarity comparison is identified, and damage cases that have the same damage locations but different damage severities with the top-ranked case must also be ranked highly. Then, the REAs of the cases within the same damage type are calculated and in turn, how much the REAs are affected by the damage level for each individual damage location in the group is computed as well. Based on the rate of changes of the REAs, the value of the elastic modulus that makes the REA zero can be approximately found by various ways ranging from simple interpolation or extrapolation to various regression or optimization methods:

(9) $$g_{i}=\frac{s_{i}-h}{h}={\left|\begin{array}{cc} \begin{array}{ccc} \frac{z_{i,1}-WI_{1}^{d}}{WI_{1}^{d}} & \frac{z_{i,2}-WI_{2}^{d}}{WI_{2}^{d}} & \cdots \end{array} & \begin{array}{ccc} \frac{z_{i,j}-WI_{j}^{d}}{WI_{j}^{d}} & \cdots & \frac{z_{i,n}-WI_{n}^{d}}{WI_{n}^{d}} \end{array} \end{array}\right|}^{T},$$

(10) $$g_{i,j}=\frac{z_{i,j}-WI_{j}^{d}}{WI_{j}^{d}}=\frac{\frac{f_{i,j}^{*}-f_{j}}{f_{j}}-\frac{f_{j}^{d}-f_{j}^{d-1}}{f_{j}^{d-1}}}{\frac{f_{j}^{d}-f_{j}^{d-1}}{f_{j}^{d-1}}}=\frac{f_{i,j}^{*}-f_{j}^{d}}{f_{j}^{d}-f_{j}},$$

(11) $$r_{i}=\frac{1}{n}\sum_{j=1}^{n}g_{i,j},$$

where ***g_i_*** is the rate of errors in detection (RED) of the *i*th DEV for the damage, *g_i,j_* is *j*th element of it, and *r_i_* is the rate of errors on average (REA) for the *i*th DEV.

### 2.4. Damage Scenarios and Meta-Modelling

An essential prerequisite task to perform all these processes heretofore described is to establish damage scenarios because they are form the guideline to generate the DEV and DEM for the similarity comparison. Basically, there are two considerations. The first is which locations, i.e., members or elements of a structure, are selected as damaged, and another is how severe the individual damages are. From a a fundamental point of view, there are an infinite number of cases for the two factors, so a reasonable way to effectively organize them is required. To solve such issues, there are many available DOEs, such as full factorial, central composite, Box–Behnken, Latin square, and Latin hypercube method, and Cavazzuti (2013) described them in detail [28].

The matter of which method is more proper depends on the characteristics of the structure, e.g., the number of total members or damageable parts. In this paper, the full factorial method is considered as the priority since it is known as the most intuitive and understandable way, so it is likely to be suitable for simple examples for verification. Briefly speaking, this method provides all possible combinations with considered factors and their levels, so the total number of samples is the number of the levels raised to the power of the number of the factors. In this paper, the latter corresponds to the number of elements while the former corresponds to the number of damage severities (or damage levels). To be more specific, let uss assume that there is a structure consisting of *u* elements, and the damage severities per individual elements can be classified into *v* different levels, including the intactness. Then, the total number of damage scenarios except the undamaged condition is calculated according to the full factorial method, as shown in Equation (12). With a view to the reduction of the total number of scenarios for simplicity, additionally assuming that damage can occur at maximum *w* locations at the same time, the total damage cases are calculated as Equation (13):

(12) $$m=v^{u}-1,$$

(13) $$m=\sum_{l=1}^{w}\left({}_{u}C_{l}v^{l}\right)-1,$$

where *m* is the total number of designed damage conditions, *u* is the total number of elements (or members) of a structure, *v* is the number of damage severities, *w* is the maximum number of damaged locations, and *l* is the number of damaged locations from 1 to *w*.

On the other hand, elements of the DEM are basically calculated from the pre-updated FE model in accordance with every single damage scenario. However, it would be time-consuming due to the complexity of an offshore structure. One alternative method to reduce the computational cost in computation is a meta-model, i.e., the surrogate model. There are various metamodeling methods used in various fields, such as the neural network, and one of the most prevalent and traditional techniques is the response surface method (RSM). The method has also been employed for SHM ranging from simulation model updating [29,30,31,32,33,34] to damage identification [35,36,37,38,39,40,41,42,43,44] in many studies. In general, this approach approximately formulates a mathematical relation, especially polynomials, matching inputs to outputs. Therefore, how well the input data represent the domain space of interest and which type of math function is chosen to present the potential characteristics of a system are key factors to ensure the correctness of a meta-model.

## 3. Verification

### 3.1. Example Structure: A Portal Frame

**Figure 4:**
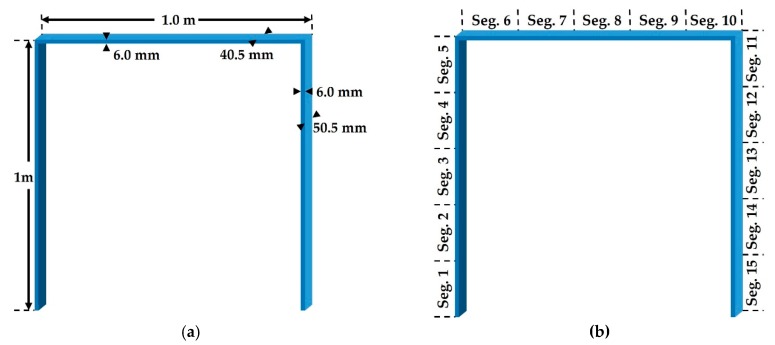
Portal Frame: (**a**) Specification; (**b**) Segments [45].

**Figure 5:**
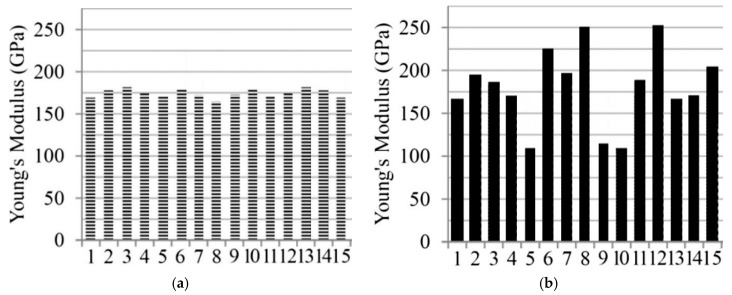
Distribution of the Young’s modulus: (**a**) Updated model F; (**b**) Updated model FMS1 [45].

This paper validates the proposed method for the 2D portal frame structure illustrated in Figure 4, referring to a recent study conducted by Umar et al. [45]. In the reference, modal tests were performed in different damage cases as well as the intact state, and respective natural frequencies from the first to fourth mode were measured. The elastic modulus of individual segments suitable for the measured dynamic characteristics was figured out by two steps of the response surface methods in sequence. In detail, the reference models, i.e., the baseline models, were primarily updated under the undamaged condition. The models were named as F and FMS1 in the reference. Model F was made based on only the measured natural frequencies whereas model FMS1 was obtained based on both the frequencies and mode shapes. Then, another RSM process was started from the reference state models for damage detection. In the entire procedure, all the FE models were comprised of 2D elements. Especially, it is interesting that the Young’s modulus of model F is symmetrically distributed as seen in Figure 5 [45], and the mean error of the natural frequencies for the measured data is much smaller than the other models.

Unlike the reference, on the other hand, the beam188 element in ANSYS Mechanical APDL was utilized to model the structure in this paper. The elastic modulus of each segment was identified using the genetic algorithm (GA) with MATLAB. The feasible region was bounded within 150 MPa to 220 MPa, and the objective function was to minimize the root mean square of the error rates of the natural frequencies as shown in Equation (14). Considering the symmetry of model F, as seen in Figure 5, the baseline models were both symmetrically and independently optimized under the intact condition in this paper. Afterwards, the cosine similarity-based damage detection was carried out for the numerical and experimental results. Table 1 shows the measured natural frequencies [45], updated natural frequencies under the undamaged condition, and the error rates per mode. The symmetric model has relatively large errors compared to the independent model. However, it does not mean that the independent model is more precise because it is just a natural result and, in general, the perspective of optimization, because more variables provide better convergence. Table 2 shows the optimum values of the elastic modulus of every segment:

(14) $$\min RMS_{ERROR}=\sqrt{\frac{1}{4}\overset{4}{\underset{j=1}{\sum }}\left[{\left(\frac{f_{j}^{s}-f_{j}^{m}}{f_{j}^{m}}\right)}^{2}\right]},$$

where *f_j_^s^* and *f_j_^m^* are the simulated and measured natural frequency at *j*th mode, respectively.

### 3.2. Numerical Validation

Two DEMs were constructed based on the updated models. The total number of designed damage cases (*m*) was 39,824 with *v* = 3 (the damage level of 7.5%, 15%, and 22.5%) and *w* = 3 (the maximum number of damaged locations), as shown in Equation (13). The damage level was defined as Equation (15):

(15) $$Damage\;level=100\times \frac{E_{DS}-E_{IS}}{E_{IS}}\left(\%\right),$$

where *E_DS_* is the elastic modulus of any damaged segment, and *E_IS_* is the elastic modulus of the segment in the intact state. In order to validate the numerical process of similarity comparison, three damage states were given as seen in Table 3. Table 4 shows the top five cases and corresponding cosine similarities of the independently updated reference model for the three damage states. As a result, it was shown that the proposed method detects all three damage conditions.

### 3.3. Experimental Verification

#### 3.3.1. Verification Process

**Figure 6:**
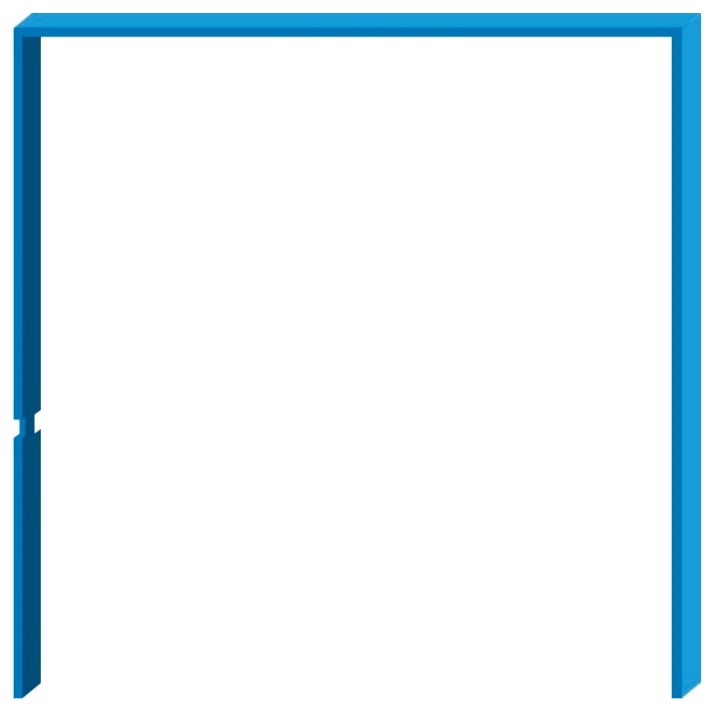
Example of a saw cut [45].

**Figure 7:**
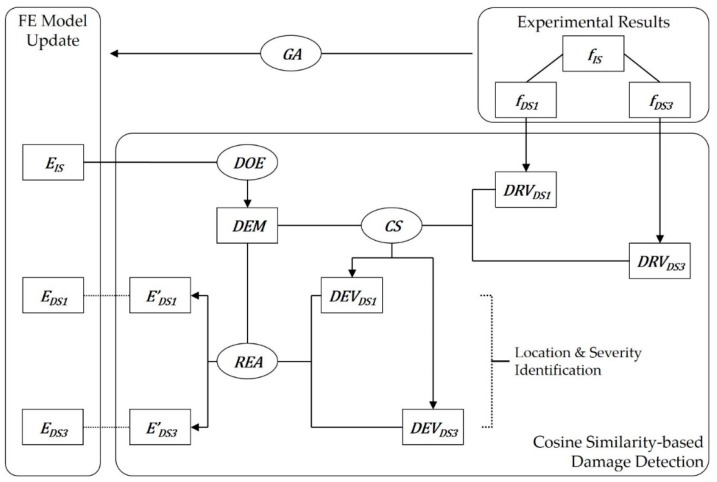
Flowchart of experimental verification for the portal frame model.

Table 5 shows the two damage states inclusive of both single and multiple damages [45] and the corresponding range of damage cases of DEM in this study. As seen in Figure 6 [45], the damages were applied as saw cuts somewhere in the segments in Table 5, so no one knows the only and absolute answer for the damage state due to inevitable uncertainties. The best way for verification is to use exact information, such as widths, depths, and locations of the saw cuts on the coordinate system, but there is no such detail in the reference. Thus, values of the Young’s modulus of each damaged segment should be evaluated as shown in Equation (14) in this paper. Considering the damages, constraints were set to allow the elastic modulus of the damaged segments to be up to 50% lower than those under the intact condition in Table 2. The lessened elastic modulus searched for by the GA and the reduction rates are listed in Table 6. The reduction rates mean the damage severity corresponding to the damage level in DRV and DEM in this study. Moreover, DRVs were calculated using Equation (7) from the natural frequencies in Table 1 and Table 7, which lists the natural frequencies in the test from [45], and in turn, the cosine similarities for an individual damage case were computed. This entire procedure is illustrated in Figure 7. Although there are four available modal data from the experimental results, it was shown that there is a relatively large error between the natural frequencies of the measurement and the updated models in the fourth mode as seen in Table 1. Hence, damage detection was conducted on not only all available modal data but only the first to the third modal data. Therefore, a total of three detection results within the top five ranked in cosine similarity were comparably analyzed, as shown in Table 8, Table 9 and Table 10.

#### 3.3.2. Result of Cosine Similarity Comparison and Damage Detection

**Figure 8:**
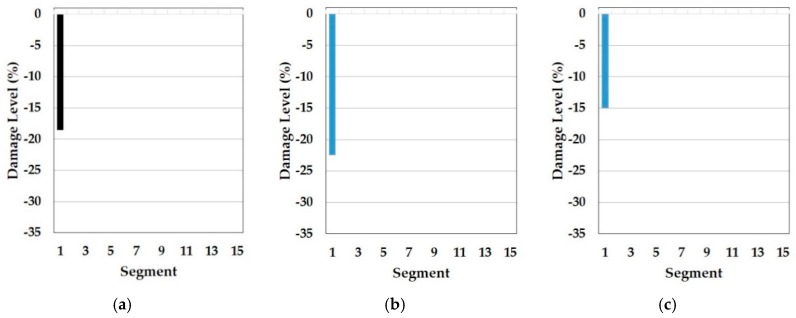
Symmetric baseline model under DS1 (blue color is correct segment whereas orange color is incorrect segment): (**a**) Predicted damage from the GA (black color); (**b**) top one damage case using the first to fourth natural frequencies; (**c**) top two damage case using the first to fourth natural frequencies.

**Figure 9:**
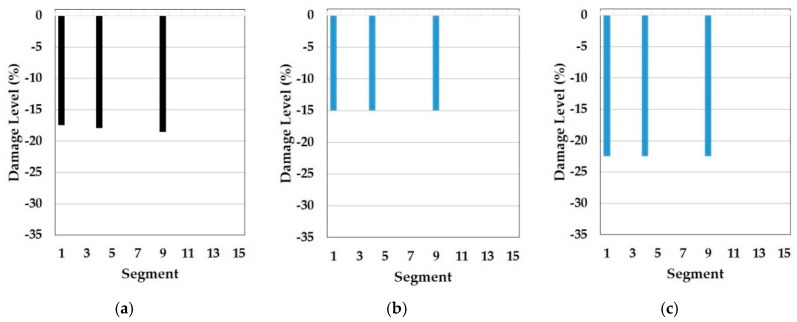
Symmetric baseline model under DS3 (blue color is correct segment whereas orange color is incorrect segment): (**a**) Predicted damage from the GA (black color); (**b**) top one damage case using the first to fourth natural frequencies; (**c**) top two damage case the first to fourth natural frequencies.

**Figure 10:**
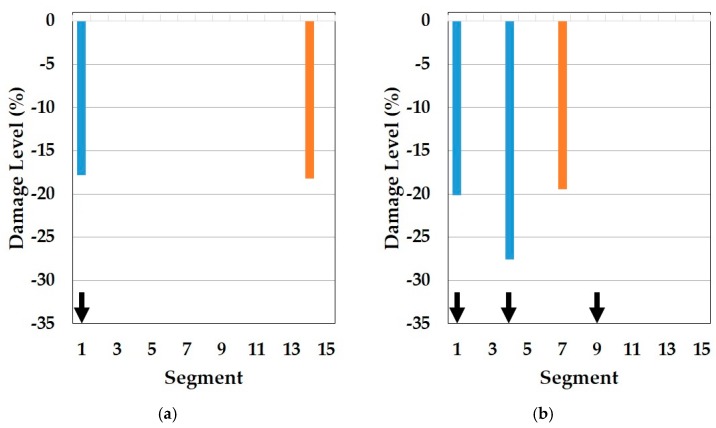
Result of damage detection with updated model F in the reference (blue color is correct segment whereas orange color is incorrect segment): (**a**) DS1; (**b**) DS2.

**Figure 11:**
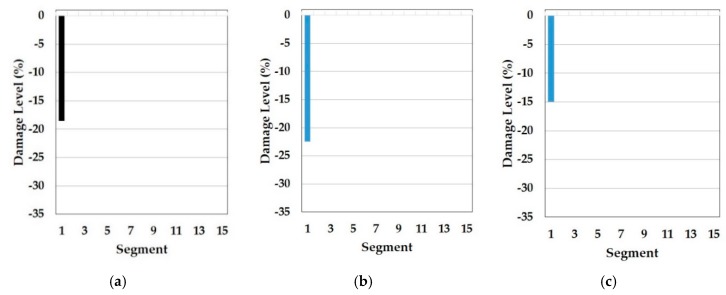
Symmetric baseline model under DS1 (blue color is correct segment whereas orange color is incorrect segment): (**a**) Predicted damage from the GA (black color); (**b**) top one damage case using the first to third natural frequencies; (**c**) top two damage case using the first to third natural frequencies.

**Figure 12:**
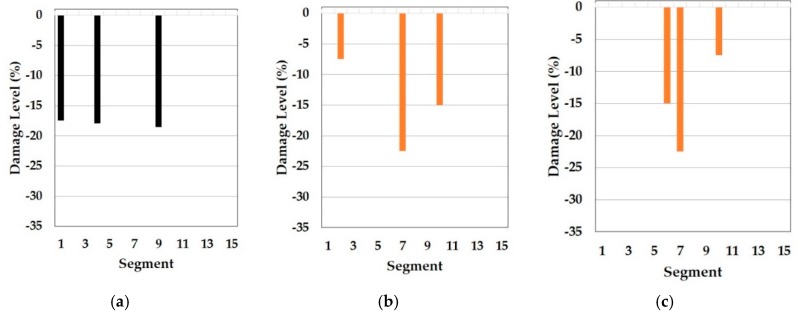
Symmetric baseline model under DS3 (blue color is correct segment whereas orange color is incorrect segment): (**a**) Predicted damage from the GA (black color); (**b**) top one damage case using the first to third natural frequencies; (**c**) top two damage case using the first to third natural frequencies.

**Figure 13:**
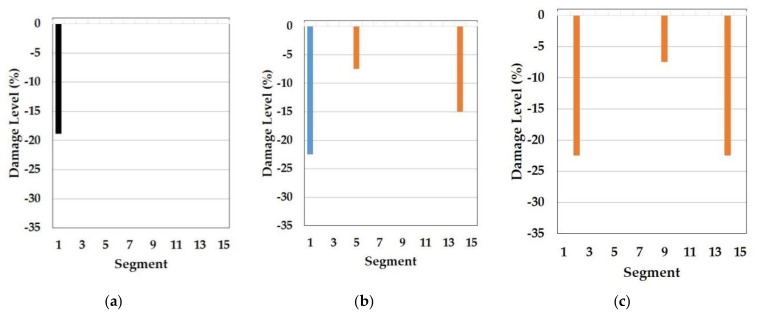
Independent baseline model under DS1 (blue color is correct segment whereas orange color is incorrect segment): (**a**) Predicted damage from the GA (black color); (**b**) top one damage case using the first to fourth natural frequencies; (**c**) top two damage case using the first to fourth natural frequencies

**Figure 14:**
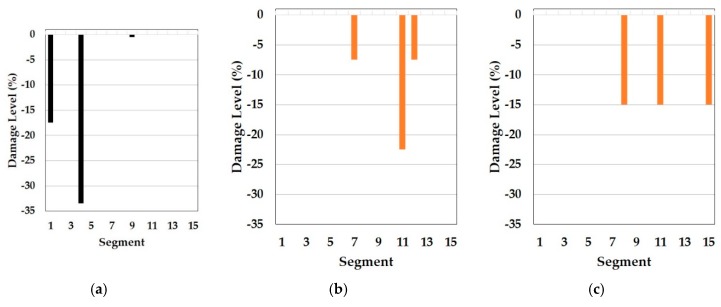
Independent baseline model under DS3 (blue color is correct segment whereas orange color is incorrect segment): (**a**) Predicted damage from the GA (black color); (**b**) top one damage case using the first to fourth natural frequencies; (**c**) top two damage case the first to fourth natural frequencies.

In this part, the results of the cosine similarity are listed in Table 8, Table 9 and Table 10. In particular, the highest five damage scenarios in cosine similarity rankings were enumerated. Among the top five cases in the tables, the two top-ranking damage cases are regarded as the results of the cosine similarity-based damage detection in this paper. Figure 8, Figure 9, Figure 10, Figure 11, Figure 12, Figure 13 and Figure 14 comparatively depict a bar-type graph. In all the figures below, plot (a) presents the predicted elastic modulus under the damaged states using the GA, and plot (b) and (c) display the top one and two damage cases in cosine similarity, respectively. The blue bars mean correctly detected results whereas the red bars mean false detection.

First, Table 8 displays the results of cosine similarity when the symmetric baseline model was used with the first to fourth natural frequencies. According to the table, there are suitable damage cases corresponding to the damages in the high ranking. To explain, damage case 2 and 3 ranked as first and second for DS1 and damage case 1787, 1800, and 1789 ranked as first, second, and fourth for DS3 are in the appropriate range of damage scenarios out of the DEM as explained in Table 5. Figure 8 and Figure 9 result from Table 8. To be specific, damage locations for both DS1 and DS3 were exactly identified. Furthermore, the top-ranked damage scenarios were correctly matched to all the damage states DS1 and DS3. It epitomizes the merit of this method in the accuracy of damage detection. In addition to this, the damage levels figured out by the GA were also placed between the damage levels of the first and the second damage cases for both DS1 and DS3, which shows the possibility that damage severity would be evaluated more accurately by the RED or REA although more studies are needed to find the most appropriate method, such as regression and inter- or extrapolation, as well as proper weighting factors among the high ranked damage scenarios.

On top of that, these cases could be comparable to the results of model F in the reference. In fact, these are the only possible pairs for result comparison with the reference, as natural frequencies ranging from the first to fourth, except the mode shapes, were utilized for the model update and for damage detection. Thus, the results correspond to each other. Specifically, the *Y*-axis in Figure 10 was modified from the stiffness reduction factor (SRF) in the reference, which is 1 − E’/E, where E and E’ is the Young’s modulus before and after damage, respectively, to the damage level, which is expressed as a percentage with the opposite sign of the SRF. With respect to the damage levels, in fact, a direct comparison would not make sense because of the different element types of FE models as well as different optimization algorithms used for each other. Despite the limitation, the performance of the damage detection method presented in this paper is obviously noticeable even only for the identification of damage locations. In comparison to Figure 10a, showing that the fault damage was detected at segment 14 under DS1, for example, Figure 8b,c represents that this method figured out the correct damage location without an overestimation of damaged places. Moreover, Figure 9b,c show that the proposed method identified all the multiple damaged segments accurately while, in the reference, the damage on segment 7 was falsely judged instead of segment 9, which is truly damaged under DS3 in Figure 10b. This indicates the contribution of the proposed method to accurate global damage identification.

Meanwhile, Table 9 represents the top five damage cases with the first to third natural frequencies of the symmetric baseline model and Figure 10 and Figure 11 describe the two detected damage cases among the five, respectively. In this approach, the result only on DS1 makes sense because there are no damage cases suitable for DS3 in the top five and the order of the rankings is different compared to Table 8, despite the same baseline model. The reason for this failure is the different range of orders of natural frequencies used to compute the cosine similarity. While the baseline model (E_IS_) and the two damaged models (E_DSx_) were optimized based on the first to fourth natural frequencies by using the GA, the similarity was calculated except for the fourth frequency. The difference leads to the error of dot products in the calculation of the cosine similarity since all elements out of the two frequency vectors play an equal role in the operator. Therefore, it is required that the same orders of natural frequencies are used to update the baseline model and in the calculation of cosine similarity to detect multiple damages accurately. Conversely, there is a question about why the damage scenarios out of the reasonable range were highly ranked in the case of DS1. One possible cause is the damage location. Segment 1 is most close to one of the fixed points, which has the most to do with the first mode and natural frequency. That is, the damage near the points would be less sensitive to higher natural frequencies. In fact, it would be a fundamental phenomenon in general structure dynamics rather than a special issue in this method. Therefore, it is evidence that this method provides convincing outcomes.

On the other hand, there is no damage scenario closely involved with the two damage states in the top five as seen in Table 10. Compared to Table 8, Table 10 comes from the independent baseline model while the same range from the first to fourth natural frequencies was utilized to make the baseline models and similarity estimation. That is, the symmetrically updated baseline model is better than the independently updated model in this example. However, it does not mean that symmetry is always better. Assumedly, this is because the portal frame structure in the test is symmetrical in the distribution of the elastic modulus. It is also supported by the fact that the updated model F, which has the least mean error in natural frequencies in the reference paper, showed a tendency of a vertical symmetric distribution of the Young’s modulus, as Figure 5 mentioned before. This means that the accuracy of this method relies on a good agreement between the baseline model and the real structure. Therefore, we know that one of the important factors in this method is how much the baseline model precisely reflects the real structure.

## 4. Conclusions

Aiming at contributing to a more applicable real-time SHM offshore, this research proposed a multiple damage detection method using cosine similarity of the rate of change of natural frequencies. To sum up, the process is comprised of three parts: Damage individualization, damage recognition, and damage identification. A damage estimation matrix is constructed from a preliminarily updated FE model using DOE and modal analysis in the first stage. In the recognition process, the rate of change of natural frequencies from OMA based on sensor data during operating is normalized and utilized as a normalized warning index. When the index becomes larger than a threshold, it is judged that damage happens, and subsequently, the damage reflection vector is generated in this state. Ultimately, cosine similarity between the damage estimation matrix and the damage reflection vector is computed, and finally the most similar damage cases among the vector sets of the estimation matrix are identified in the ranking of similarity.

Therefore, the best benefit of this method is that any extra task and professional knowledge is not required at all for workers in the real offshore field to perceive the occurrence, locations, and severities of damage because a damage warning and ranking of most possible damage cases is provided in the damage recognition and identification and complicated and numerous computational tasks in the damage individualization are completed in the very initial stage of operation. Such a simple and intuitive system to treat damage would absolutely improve the applicability and usability of SHM in offshore operations.

For verification, the portal frame model from a reference was used. Two types of damages, DS1 and DS3, were considered: DS1 is single damage and DS3 is multiple damages. Two different baseline FE models, a symmetric and independent model, were established by the GA. In the numerical validation, the first ranked damage cases with cosine similarities of 1 had identical damages with the input of damages for both the DS1 and DS3. Therefore, the correctness of the numerical process was proven. Moreover, there were three examples for the experimental data: Symmetric model with the first to fourth natural frequencies, independent model with the same natural frequencies, and symmetric model with the first to third natural frequencies. For the first case of experimental verifications, the two top candidates of scenarios accurately predicted damaged locations on segment 1 for DS1 and on segments 1, 4, and 9 for DS3. Such accuracy was better than the performance of the damage detection technique in the reference. In terms of damage severity, an 18.57% reduction of the Young’s modulus at segment 1 under DS1 was placed within −15% and −22.5%, which were the damage levels of the top two scenarios under DS1. Damage severities of −17.45%, −17.95%, and −18.55% for segment 1, 4, and 9, respectively, under DS3 were also in the range between −15% and −22.5% of the damage levels of the two top cases under DS3. These indicate that there is still room for improvement of the preciseness in this way by using the rate of errors on average (REA) based on the similarity results. On the other hand, the other examples provided insight into important factors on this approach. In the case of the independent model, DS1 was correctly identified whereas DS3 was falsely detected. This indicates that the precision of this method depends on the correctness of a preliminarily updated model as all the inverse SHM methods. Plus, the last example completely misestimated both DS1 and DS3. The lesson of this is that the utilization of the same orders of natural frequencies both in updating a baseline model and in computing the cosine similarity is the way to obtain accurate results for damage detection in this method.

As future works, there are several directions to further develop this method. The first is to conduct a comparative study to examine which method among the various similarity methods is most appropriate. One possible criteria for this is to find the similarity method capable of making the values of similarity more distinguishable from one another than now, as the difference of the cosine similarity values between consecutive loading conditions in order of the ranking was smaller than 10 to 4 in this paper, which may seem less significant, compared to the whole range of cosine similarity from 0 to 1. Added to the similarity investigation, secondly, performance evaluation of this technique for other unavoidable types of structural damages that arise in offshore structures, for instance, the change of sections owing to degradation and corrosion in saline water and the change of masses due to ocean biofouling, should be researched to extend the applicability of this method to real offshore structures. This could be the beginning of a study to identify typical specific attributes corresponding to the inevitable damage types of offshore structures, which, in turn, would greatly help to perform the next investigation, such as on-the-spot inspection, repair of failures, and new baseline model updates. Thirdly, tests on which kind of baseline FE models are more suitable for this baseline model-dependent technique would be one possible future task because the model update using mode shapes or using both, but not only natural frequencies, results in different FE baseline models. The distinction would lead to different results, as well. Lastly, our own experiments on more complex structures, such as a prototype of a jacket structure, might be carried out before applying this presented technique to a real structure in the offshore field since there are many implications in sensing and signal processing as mentioned previously. Such a test would provide more practical findings and broader perspectives than what was done in this paper for this comprehensive SHM method, ranging from measurement, signal processing, OMA, damage identification, and on-the-spot inspection to repairs and new model updates.

## Figures and Tables

**Table 1:** Natural frequencies in the undamaged state [45] and the corresponding updated results.

	Measured	Symmetric Model		Independent Model	
	NF (Hz)	NF (Hz)	Error Rate (%)	NF (Hz)	Error Rate (%)
1	4.53	4.53	0.00669	4.53	0.00104
2	17.6	17.59	–0.07901	17.59	–0.03555
3	28.3	28.28	–0.06045	28.3	0.00899
4	30.7	30.67	–0.10317	30.72	0.05276

**Table 2:** Optimal elastic modulus of the intact portal frame (GPa).

Segment	1	2	3	4	5	6	7	8	9	10	11	12	13	14	15
E_sym_	186.0	205.9	172.2	202.7	174.4	180.1	165.9	178.6	165.9	180.1	174.4	202.7	172.2	205.9	186.0
E_ind_	186.9	204.9	179.4	202.1	178.5	201.3	177.1	163.4	213.5	173.2	152.9	195.9	165.3	207.7	181.8

**Table 3:** Damage location and severity for numerical validation.

State	Segment Number	Damage Level
DC1	1	−22.5%
DC3	1	−22.5%
	4	−22.5%
	9	−15.0%

**Table 4:** Damage cases ranked in the top five and the corresponding cosine similarities of the independently updated baseline model.

	DC1				DC3			
	CS	Case	Seg. ^1^	Level ^2^	CS	Case	Seg. ^1^	Level ^2^
**1**	**1.000000**	**3**	**1**	**−22.5**	**1.000000**	**1799**	**1**	**−22.5**
							**4**	**−22.5**
							**9**	**−15**
2	0.999997	3251	1	−22.5	0.999996	12561	9	−15
			11	−7.5			11	−22.5
			14	−15			15	−7.5
3	0.999971	5113	2	−22.5	0.999903	9456	5	−15
			9	−7.5			8	−15
			14	−7.5			15	−22.5
4	0.999946	3242	1	−15	0.999878	9278	5	−22.5
			11	−7.5			7	−22.5
			14	−15			15	−15
5	0.999915	3255	1	−22.5	0.999818	3022	1	−7.5
			11	−15			9	−22.5
			14	−22.5			15	−7.5

^1^ Seg. is segment number. ^2^ Unit of level is percent (%).

**Table 5:** Damage state [45] and the range of corresponding damage cases.

State	Segment	Number of Cuts	Range of Damage Cases of DEM
DS1	1	5	1–3
DS3	1	5	1774–1800
	4	5	
	9	4	

**Table 6:** GA results: elastic modulus (E_DSx_) and damage severities ^1^ of damaged segments (GPa, %).

Model	Symmetric Model						Independent Model					
Segment	1		4		9		1		4		9	
Units	GPa	%	GPa	%	GPa	%	GPa	%	GPa	%	GPa	%
IS ^2^	186.0	-	202.7	-	165.9	-	186.9	-	202.1	-	213.5	-
DS1	151.5	−18.57	-	-	-	-	151.7	−18.85	-	-	-	-
DS3	153.5	−17.45	166.3	−17.95	135.1	−18.55	154.3	−17.46	134.5	−33.48	213.4	−0.079

^1^ Damage severity = 100 × (E_DSx_ − E_IS_)/E_IS_ like Equation (15). ^2^ IS means the intact state.

**Table 7:** Natural frequencies of the damaged frame (Hz) [45].

Mode	DS1	DS3
1	4.42	4.41
2	17.5	17.1
3	27.9	27.6
4	30.5	30.4

**Table 8:** The top five damage cases in cosine similarity with the first to fourth natural frequencies of the symmetric baseline model.

	DS1				DS3			
	CS	Case	Seg. ^1^	Level ^2^	CS	Case	Seg. ^1^	Level ^2^
E_DSx_	-	-	1	−18.57	-	-	1	−17.45
							4	−17.95
							9	−18.55
1	**0.999934**	**2**	**1**	**−15**	**0.999880**	**1787**	**1**	**−15**
							**4**	**−15**
							**9**	**−15**
2	**0.999925**	**3**	**1**	**−22.5**	**0.999788**	**1800**	**1**	**−22.5**
							**4**	**−22.5**
							**9**	**−22.5**
3	0.999785	160	1	−22.5	0.999779	1735	1	−15
							4	−22.5
			14	−7.5				
							7	−7.5
4	0.999764	1929	1	−22.5	**0.999646**	**1789**	**1**	**−15**
			4	−7.5			**4**	**−22.5**
			14	−22.5			**9**	**−7.5**
5	0.999755	52	1	−22.5	0.999511	3901	2	−22.5
							4	−15
			2	−7.5				
							9	−7.5

^1^ Seg. is segment number. ^2^ Unit of level is percent (%).

**Table 9:** The top five damage cases in cosine similarity with the first to third natural frequencies of the symmetric baseline model.

	DS1				DS3			
	CS	Case	Seg. ^1^	Level ^2^	CS	Case	Seg. ^1^	Level ^2^
E_DSx_	-	-	1	−18.57	-	-	1	−17.45
							4	−17.95
							9	−18.55
1	**0.999995**	**3**	**1**	**−22.5**	0.999998	4643	2	−7.5
							7	−22.5
							10	−15
2	**0.999995**	**2**	**1**	**−15**	0.999984	10105	6	−15
							7	−22.5
							10	−7.5
3	0.999994	1060	1	−15	0.999975	2626	1	−15
			2	−22.5			7	−22.5
			5	−7.8			13	−7.5
4	0.999967	2081	1	−15	0.999958	2595	1	−15
			5	−7.5			7	−7.5
			10	−15			12	−22.5
5	0.999955	2072	1	−7.5	0.999957	2822	1	−22.5
			5	−7.5			8	−15
			10	−15			13	−15

^1^ Seg. is segment number. ^2^ Unit of level is percent (%).

**Table 10:** The top five damage cases in cosine similarity with the first to fourth natural frequencies of the independent baseline model.

	DS1				DS3			
	CS	Case	Seg. ^1^	Level ^2^	CS	Case	Seg. ^1^	Level ^2^
E_DSx_	-	-	1	−18.85	-	-	1	−17.46
							4	−33.48
							9	−0.079
1	0.999970	2198	1	−22.5	0.999839	11,500	7	−7.5
			5	−7.5			11	−22.5
			14	−15			12	−7.5
2	0.999951	5113	2	−22.5	0.999744	12,155	8	−15
			9	−7.5			11	−15
			14	−22.5			15	−15
3	0.999950	3242	1	−15	0.999607	4964	2	−7.5
			11	−7.5			8	−15
			14	−15			15	−15
4	0.999908	3255	1	−22.5	0.999570	2951	1	−15
			11	−15			9	−22.5
			14	−22.5			12	−15
5	0.999904	3251	1	−22.5	0.999540	12,158	8	−15
			11	−7.5			11	−22.5
			14	−15			15	−15

^1^ Seg. is segment number. ^2^ Unit of level is percent (%).
